# PNOC009: Convection-enhanced delivery of liposomal irinotecan in patients with newly diagnosed diffuse intrinsic pontine glioma

**DOI:** 10.1093/noajnl/vdaf093

**Published:** 2025-05-13

**Authors:** Sabine Mueller, Cassie Kline, Alex Y Lu, Raoull Hoogendijk, Eva Wembacher-Schroeder, Anuradha Banerjee, Alyssa T Reddy, Shannon Raber, Carly Hoffman, Schuyler Stoller, Michael Prados, Annette Molinaro, Nalin Gupta

**Affiliations:** Department of Pediatrics, University of Zurich, Zurich, Switzerland; Department of Pediatrics, University of California, San Francisco, San Francisco, California, USA; Department of Neurology, University of California, San Francisco, San Francisco, California, USA; Department of Neurological Surgery, University of California, San Francisco, San Francisco, California, USA; Department of Pediatrics, Perelman School of Medicine, University of Pennsylvania, Philadelphia, Pennsylvania, USA; Division of Oncology, Children’s Hospital of Philadelphia, Philadelphia, Pennsylvania, USA; Department of Neurological Surgery, University of California, San Francisco, San Francisco, California, USA; Department of Neurology, Brain Tumor Center, Erasmus MC Cancer Institute, University Medical Center Rotterdam, Netherlands; Princess Máxima Center for Pediatric Oncology, Utrecht, The Netherlands; Brainlab AG, Munich, Germany; Center for Cancer and Blood Disorders, University of California, San Francisco, San Francisco, CA, USA; Department of Pediatrics, University of California, San Francisco, San Francisco, California, USA; Department of Neurology, University of California, San Francisco, San Francisco, California, USA; Department of Pediatrics, University of California, San Francisco, San Francisco, California, USA; Department of Pediatrics, University of California, San Francisco, San Francisco, California, USA; Department of Neurology, University of California, San Francisco, San Francisco, California, USA; Department of Pediatrics, University of California, San Francisco, San Francisco, California, USA; Department of Neurological Surgery, University of California, San Francisco, San Francisco, California, USA; Department of Neurological Surgery, University of California, San Francisco, San Francisco, California, USA; Department of Pediatrics, University of California, San Francisco, San Francisco, California, USA; Department of Neurological Surgery, University of California, San Francisco, San Francisco, California, USA

**Keywords:** convection-enhanced delivery, DIPG, nano liposomal irinotecan

## Abstract

**Background:**

Median survival in patients with diffuse intrinsic pontine glioma (DIPG) varies between 11 months for children and 20 months for adults. There is no standard of care treatment other than radiation therapy. This study aimed to determine the safety, tolerability, and distribution of nanoliposomal irinotecan (nal-IRI) delivered via CED in patients with DIPG.

**Methods:**

Newly diagnosed DIPG patients > 2 years were eligible for enrollment. Dose level (DL) 1 and 2 were completed (DL1 = 2 mL of nal-IRI [20 mg/mL] DL2 = 3 mL of nal-IRI [20 mg/mL]). Nal-IRI was co-infused with gadoteridol via CED to achieve maximal tumor coverage. The distribution of infusate mixture was monitored with real-time magnetic resonance imaging (MRI). The study employed an accelerated dose escalation approach and transitioned to a conventional 3 + 3 design based on predefined rules. The study was terminated prematurely due to the discontinuation of drug supply by industry partner.

**Results:**

Six patients (median age 10 years, range 5–40) underwent a total of 13 treatments (median 2, range 1–5/patient). Four grade 3 adverse events, (muscle weakness *n* = 2, dysarthria *n* = 1, and gait disturbance *n* = 1) were observed, including one dose-limiting toxicity (muscle weakness). Mean tumor volume prior to the first CED treatment was 28.9 ± 8.8 cm^3^ with a mean tumor coverage per treatment of 35.3% ± 17.6. Twelve-month overall survival (OS) was 67% (95% CI 38–100).

**Conclusions:**

Repeated CED of nal-IRI in patients with DIPG demonstrated an acceptable risk profile with reasonable tumor coverage. Additional investigations utilizing this strategy should be evaluated in a larger patient cohort to determine efficacy.

**Key Points**

- Pre-clinical studies utilizing CED of nal-IRI have shown encouraging results.
- PNOC009 showed repeated CED with nal-IRI was tolerable.
- Repeated CED achieves reasonable tumor coverage, supporting larger cohort evaluation.

**Importance of the Study**

Patients with diffuse intrinsic pontine glioma (DIPG) desperately need new treatment options. Nanoliposomal irinotecan (nal-IRI) demonstrated promising effectiveness with extended drug exposure in pre-clinical DIPG models. Convection-enhanced delivery (CED) delivers drugs directly to the tumor at higher local concentrations by circumventing the blood–brain and blood–tumor barriers. PNOC009 showed that CED of nal-IRI in DIPG patients is well tolerated. In addition, PNOC009 complements previous studies showing that repeated CED in DIPG patients is safe. Lastly, by incorporating the concurrent administration of gadoteridol, PNOC009 offers insights into the volumetric drug distribution of nal-IRI. The results of PNOC009 reinforce earlier studies utilizing CED in the DIPG population and provide additional safety and feasibility data for repeated catheter placement and infusions.

Patients diagnosed with a diffuse intrinsic pontine glioma (DIPG), have a median survival of only 11 months in children and around 20 months for adults.^1–3^ Presently, the standard treatment is limited to focal, fractionated radiotherapy. Key obstacles to successful treatment are the blood–brain and blood–tumor barrier, which prevents potentially effective drugs from reaching the tumor in sufficient concentrations.^4^ Convection-enhanced delivery (CED) offers a solution by allowing direct delivery to the brain interstitial space resulting in maximal drug exposure to target tumor cells.^4–6^ Irinotecan has shown promising efficacy in laboratory models and patient-derived brainstem xenografts of DIPG, particularly when delivered directly into the tumor rather than systemically.^7,8^ Preclinical studies in a glioblastoma model demonstrated that CED of encapsulated irinotecan in liposomes (nal-IRI) significantly improved survival compared to CED with free irinotecan and controls.^9^ Additionally, nal-IRI exhibited prolonged retention and an extended half-life in brain tumor tissue relative to free irinotecan. These considerations are critical in strategies such as CED, which require repeated catheter placements and thus, limit frequent drug administration.^9,10^

PNOC009 is a phase 1 study exploring CED with nal-IRI alongside real-time imaging in newly diagnosed DIPG (NCT03086616). The primary aim of the trial was to evaluate the safety as well as intratumoral distribution of nal-IRI. The primary hypothesis was that repeat CED of nal-IRI after standard radiotherapy is safe and will improve overall survival (OS) in patients with newly diagnosed DIPG. Herein, we report the results of PNOC009 and the potential for the next steps with this novel drug delivery therapy.

## Patients and Methods

### Study Design

PNOC009 is an open-label, phase 1 study investigating CED of nal-IRI infused with gadoteridol and applying real-time imaging to assess infusate distribution in patients with newly diagnosed DIPG. The study enrolled at UCSF Benioff Children’s Hospital, San Francisco, United States from October 2017 to December 2018. The study was closed to accrual prematurely in September 2021 due to the discontinuation of drug supply by the sponsoring company (Ipsen).

The primary objective of PNOC009 was to assess the safety and toxicity of first-time and repeated CED of nal-IRI. Safety and toxicity assessments were conducted using the Common Terminology Criteria for Adverse Events (CTCAE) version 4.0. The study followed an accelerated titration design (ATD) as described by Simon et al.^11^ Dose-limiting toxicity (DLT) was characterized as (1) any intolerable grade 2 or any grade 3 or higher neurological toxicity related to CED procedure or nal-IRI infusion with gadoteridol or (2) any systemic treatment-related grade 3 or higher hematologic or non-hematologic toxicity (with exception of allowance for maximal medical management of nausea/vomiting/diarrhea). The DLT period was defined as 14 days after the last infusion.

Intra-patient dose modification was allowed based on toxicity and as per a priori escalation rules (Supplementary **Figure S1**). If a patient experienced grade 0 or 1 toxicity, dose escalation was permitted, while dose de-escalation occurred when a DLT. When tolerable grade 2 toxicity was observed, the dose remained unchanged. Upon the second occurrence of grade 2 or higher toxicity or first DLT during an initial treatment cycle, the study design mandated the transition to a standard 3 + 3 dose-escalation design, enrolling two additional patients at the dose level that triggered this transition. Based on this design, if two or more DLTs occur in three patients, the recommended phase 2 dose (RP2D) would be the next lower dose level. If one DLT occurred, three additional patients enrolled at the same dose level. If only one DLT was observed in six patients, dose escalation would continue. However, if more than one DLT was observed, the next lower dose would be determined as the RP2D. If no DLTs were observed, dose escalation proceeded. The intent was to treat every 4–8 weeks, as long as there was no evidence of tumor progression within the treated area or intolerable toxicity.

The secondary objective of PNOC009 was to determine the clinical efficacy of repeated administration of nal-IRI given by CED in children with newly diagnosed DIPG, as defined by OS at 12 months (OS12). OS was the duration of time from diagnosis based on imaging to time of death. Based on Cohen et al. patients newly diagnosed with DIPG treated with a combination of radiation therapy and temozolomide were expected to have an OS12 rate of 40% (SD ± 6.5%).^12^ For this study, after the inclusion of 19 patients, if 12 or more patients were alive at 12 months, the null hypothesis that OS12 is 40% would be rejected.

Lastly, as an exploratory objective, PNOC009 evaluated the magnetic resonance image-guided intratumoral drug coverage through co-infused gadoteridol distribution. This study was approved by the Institutional Review Board of the University of California, San Francisco (UCSF; CC#160816) and written informed consent and assent, as applicable, were obtained from all patients, parents, and/or guardians prior to the study entry.

### Eligibility

Patients ≥ 2 years of age with newly diagnosed DIPG on magnetic resonance imaging (MRI; defined as pontine location with diffuse involvement of at least 2/3 of the pons) were eligible for study enrollment. Histologic diagnosis was not required. Completion of radiotherapy prior to enrollment was mandated. CED treatment with nal-IRI began between 4 and 14 weeks after completion of focal radiotherapy. If a patient had received any prior chemotherapy, the last dose had to be given at least 30 days from the start of CED infusion with the exception of antibodies, which required completion of at least three half-lives. Patients were required to have a Lansky Play-Performance Scale or Karnofsky Performance Status ≥50 as well as life expectancy greater than 12 weeks measured from the date of radiotherapy completion with patients on a stable or decreasing dose of corticosteroids prior to registration. Other inclusion criteria included standard organ function requirements and adequate seizure control in patients with a history of seizures.

Patients were excluded if they had clinical or radiographic tumor progression following radiotherapy or metastasis including leptomeningeal or subarachnoid disseminated disease. Patients with tumor morphology or other imaging predicting poor tumor coverage such as significant tumor volume outside of the pons or large cysts as well as untreated symptomatic hydrocephalus were also excluded. Detailed inclusion and exclusion criteria are provided in **Supplementary Table S1**.

Prior to enrollment, an institutional multidisciplinary tumor board confirmed eligibility based on imaging and clinical review.

### Treatment Planning

Patients who met eligibility criteria underwent a baseline brain MRI within 14 days prior to catheter placement for CED. The tumor target was selected by a multidisciplinary team, which included the study neurosurgical chair (NG) and study chairs (SM, MP, CK). A software program (Brainlab AG, Munich, Germany), was utilized to select a trajectory that avoided critical structures such as the fourth ventricle, blood vessels, and essential motor/sensory tracts. A trajectory through the middle cerebellar peduncle was chosen for 12 of 13 infusions. One trajectory used a supratentorial approach for catheter placement. To avoid reflux, repeat infusions were usually performed through a contralateral entry point. Target and entry points were optimized for each trajectory to provide maximum tumor coverage, avoid leakage along previous catheter tracts, and sequestration into necrotic/cystic tumor compartments. Screenshots of the planned trajectory underwent review and approval by the study neurosurgeon, study chairs, and treating neuro-oncologist. All catheter placements and initiation of CED drug infusions took place in an intraoperative MRI operating room (OR), which consisted of a neurosurgical OR adjacent to a 3T MRI scanner (750W, General Electric Healthcare; Waukesha, WI).

### Catheter Placement

The placement of CED infusion catheters (Brainlab Flexible Catheters) utilized a frameless image-guided stereotactic system (VarioGuide^TM^, Brainlab AG). On the first day of CED infusion, all patients underwent general anesthesia induction in the OR. For patients undergoing consecutive day 2 of CED without the need for new catheters, anesthesia was administered either in an induction room adjacent to the intraoperative MRI OR or within the OR.

During each procedure, nine fiducial markers were placed on the scalp and the head was secured using an MR-conditional head clamp (Intregra, Princeton, NJ). Patients were positioned supine with the head turned to the opposite side of the entry point. T1- and T2-weighted sequences were acquired after patient positioning and head immobilization. Obtaining navigation sequences after head immobilization prevented scalp movement and changes in head position, ensuring more precise catheter placement. A comparison of the planned trajectory and target with the actual catheter position revealed deviations ranging between 0.5 and 2 mm.

The baseline MR images obtained prior to the day of the procedure were used to pre-plan catheter trajectories. Fiducial-based registration was performed using the MR images obtained on the day of the procedure. These images were fused with the baseline MR images containing the pre-planned trajectories. The VarioGuide system was aligned utilizing the Brainlab navigation software, followed by a small skin incision and twist drill hole to accommodate the catheter anchor. The Brainlab Flexible Catheter was then placed at the pre-determined navigated depth. An intra-operative MRI was obtained to confirm catheter position prior to drug infusion.

### Liposomal Irinotecan Infusion

With each infusion, nal-IRI (20 mg/mL) was mixed with gadoteridol (ProHance®) and delivered through catheters once the planned position was confirmed. Gadoteridol was selected for its superior stability and safety compared to other contrast agents. The nal-IRI and gadoteridol co-infusion started at 1 µL/min with a gradual increase, reaching a maximum of 5 µL every 15 min under real-time imaging to a maximum of 10 µL/min. Vital signs of the patient were continuously monitored and the infusion rate was slowed if concerning vital sign changes occurred.

Repeated T1-weighted images were taken during the infusion to monitor the distribution of the infusate. The total infusion time was influenced by factors such as tumor volume, the ratio of volume of infusion (Vi) to volume of distribution (Vd), and the flow rate. The maximum volume allowed was determined by the patient dose level. Adjustments to infusion parameters were made based on real-time MRI observations. If backflow along the catheter was detected, the infusion rate could be slowed or the catheter repositioned. In cases where distribution from a specific catheter placement extended beyond the target volume, options included advancing or withdrawing the catheter along the same trajectory, stopping the infusion, and repositioning the catheter along a different trajectory.

Once a stable infusion rate was achieved, the patient was monitored in a location close to the MR scanner, either in the adjacent operating room (OR) or post-anesthesia care unit (PACU), or transferred to the intensive care unit (ICU) for completion of the infusion. When the patient was sufficiently awake to follow commands, neurologic assessments were performed hourly, tailored to the patient’s level of consciousness and age. Continuous vital sign monitoring included blood pressure checks at least every 15 min for the duration of the infusion. At the end of the infusion (maximum 48 h), additional images were obtained to assess the final volume of distribution.

The catheters were removed following the completion of the CED infusion. Repeat CED infusions occurred every 4–8 weeks in the absence of tumor progression within the treated area or toxicity. Assessment of safety and toxicity continued over a 14-day period after each CED.

### Irinotecan Distribution and Tumor Coverage

The determination of tumor volume relied on 3D volumetric T2 FLAIR signal hyperintensity, encompassing cystic/necrotic tumor compartments, assessed at baseline before CED treatment. Tumor volumetric assessment was conducted using the semi-automatic segmentation feature of iPlan Flow planning software (Brainlab AG). The segmentation results underwent verification by a neuroradiologist who was blinded to the study. The spatial distribution volume of gadoteridol (Vd Gad) was determined using a customized subtraction method. Pre- and post-infusion 3D T1-weighted MR images acquired before and after CED infusion were co-registered to establish spatial correspondence. The region of infused tissue was delineated through gross segmentation of post-infusion imaging, defining the region of interest (ROI). Additionally, an anatomical structure unaffected by the infusion was automatically segmented based on pre-infusion images. The intensity distributions of both structures were analyzed and normalized across pre- and post-infusion datasets. This normalized intensity profile was then utilized to automatically segment signal changes within the ROI that were specifically attributable to the T1 shortening effect of gadolinium.

The Vd Gad was segmented individually for each infusion in total and then normalized by calculating the ratio Vd Gad/volume of infusion (Vi). The volume of total gadoteridol distribution inside the tumor volume was calculated for each subject, representing tumor coverage, and reported as a percentage of the lesion volume.

### Statistical Analyses

All patients enrolled were included in the analyses. The study was terminated after the sixth patient completed the study as the sponsoring company discontinued the drug supply.

Adverse events and clinically significant laboratory abnormalities (meeting Grade 3, 4, or 5 criteria according to CTCAE) are summarized by maximum intensity and relationship to study intervention and drug. Grade 1 and 2 adverse events are summarized if related to study therapy.

OS was defined as the duration of time from diagnosis based on imaging to time of death. The survival time for patients without a recorded death was defined as the duration of time from diagnosis based on imaging to the off-study date. OS12 and median OS with the corresponding confidence intervals (CIs) were estimated using the Kaplan–Meier method.

Tumor volume and drug distribution are reported descriptively and limited to frequency tables and summary statistics. All statistical analyses were performed utilizing R statistical programming language version 4.3.2 with survival analyses conducted using the *survival* package.

## Results

### Patient Population and Study Treatment

**Figure 1.**
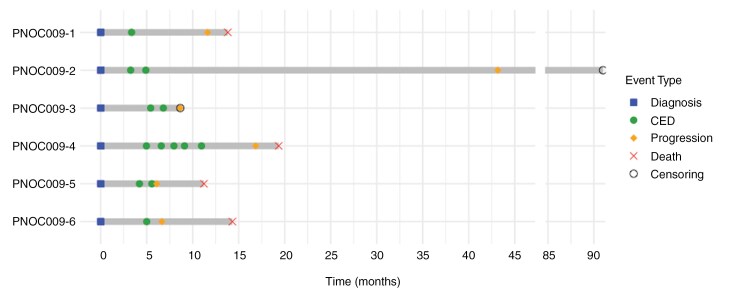
Study timeline per patient

In total, six patients (males, *n* = 2; median age 10 [range 5–40]) were enrolled between November 2017 and December 2018 (**Table 1**). All patients underwent radiotherapy prior to enrollment, averaging 2.4 ± 0.9 months between diagnosis and radiotherapy initiation as shown in **Figure 1**. One patient (PNOC009-1) underwent ventriculoperitoneal shunting before study enrollment, one patient (PNOC009-5) had a biopsy before treatment and another patient (PNOC009-2) 83.5 months after the primary diagnosis. For PNOC009-5 immunohistochemistry supported the diagnosis of DMG, demonstrating diffuse H3 K27M positivity in tumor nuclei and loss of H3 K27me3 staining in neoplastic nuclei, negative for IDH-1 mutant protein, retained ATRX expression in tumor nuclei, and negative p53. For PNOC009-2, a diagnosis of isocitrate dehydrogenase (IDH)-mutant glioblastoma was confirmed. The median time from initial diagnosis to first CED treatment was 4.5 months (range 3.3–5.4; **Figure 1**). Four patients had repeat CED treatments (range 2–5), while two patients received only one treatment (overall median 2). Administration of dose levels (DL) 1 and 2 was completed, corresponding to nal-IRI at 2 mL (20 mg/mL) for DL1 and 3 mL (20 mg/mL) for DL2. Overall, the cohort underwent a total of 13 CED treatments.

**Table 1.** Demographics and Study Timeline

Subject	Biologic sex	Age at diagnosis (years)	Total CED treatments	Dose level	dlt	Total months of follow-up	Survival status at last follow-up
PNOC009-1	F	7.5	1	1	No	13.7	Death
PNOC009-2	M	40.2	2	2	No	65.4	Alive
PNOC009-3	M	13.1	2	2-1	Yes	9.1	Lost to F/U
PNOC009-4	F	7.2	5	2	No	19.3	Death
PNOC009-5	F	5.5	2	2	No	11.2	Death
PNOC009-6	F	31.5	1	2	No	14.3	Death

Abbreviations: CED = Convection-enhanced delivery; DLT = Dose-limiting toxicity; F/U = Follow-up.

### Treatment Toxicities

The study therapy was well tolerated with a total of four grade 3 adverse events (AEs) observed in three patients (**Table 2**). Grade 3 AEs included muscle weakness (PNOC009-1; day 1 (D1); cycle 1), muscle weakness (PNOC009-3; D15; cycle 1), dysarthria (PNOC009-5; D22; cycle 2), and gait disturbance (PNOC009-5; D22; cycle 2). One grade 3 AE (muscle weakness PNOC009-3; D15; cycle 1) was considered related to the surgical intervention and therefore defined as a DLT. Grade 1 and 2 treatment-related AEs (TRAEs) were limited and consisted of facial nerve disorder left (PNOC009-3; grade 1, D15; cycle 1), facial nerve disorder right (PNOC009-3; grade 1, D9; cycle 2), glossopharyngeal nerve disorder (PNOC009-3; grade 1, D9; cycle 2), hypoglossal nerve disorder (PNOC009-3; grade 1, D9; cycle 2), headache (PNOC009-4; grade 1, D9; cycle 1), Muscle weakness (PNOC009-5; grade 2, D9; cycle 1), and scalp pain (PNOC009-6; grade 2, D8; cycle 1). PNOC009-2 experienced several grade 2 AEs (abducens nerve disorder, dizziness, fatigue, urinary retention, and decreased lymphocyte count) during cycle 1, leading to the transition of the ATD to a 3 + 3 design after the occurrence of second grade 2 AE during cycle 1. All AEs are summarized in Supplementary **Table S2**. Only one patient discontinued therapy due to TRAEs. Due to the premature termination of the study, we were not able to determine the RP2D.

**Table 2.** Summary of Grade 3 Treatment-Related Adverse Events

Toxicity	Frequency	Relatedness Nal-IRI	Relatedness surgery	Outcome	Concomitant medication
Muscle weakness (right)	2	Unrelated (*n* = 1), Possibly related (*n* = 1)	Unrelated (*n* = 1), Definitely related (*n* = 1)	Not resolved	No (*n* = 1) Dexamethasone (*n* = 1)
Dysarthria	1	Unrelated	Unrelated	Resolved	No
Gait Disturbance	1	Unrelated	Unrelated	Resolved	No

Abbreviations: **Nal-IRI = Nanoliposomal irinotecan**.

### Survival and Post-study Treatment

As of the last follow-up (15 March 2025), four patients were deceased, one patient was alive, and one was lost to follow-up. OS12 was 67% (95% CI 38–100) when treating loss to follow-up as an event at the time of occurrence. In contrast, censoring the lost patient resulted in an OS12 of 80% (95% CI, 52–100). Median OS was 14.3 months (95% CI, 13.8–upper limit not available). The study included one adult patient (PNOC009-2) later diagnosed with an IDH-mutant glioblastoma, that was alive for 91 months without progression at the last follow-up visit.

Four patients received treatment after the study period. PNOC009-1 underwent intra-arterial chemotherapy, while PNOC009-2, PNOC009-4, and PNOC009-5 received re-irradiation. Additionally, PNOC009-2 received ONC201, temozolomide, and bevacizumab. PNOC009-5 was treated with bevacizumab and a single cycle of nivolumab.

### CED Treatment and Gadoteridol Distribution

The mean infusion time across all infusions was 361.6 ± 65 min. All patients received 20 mg/mL of liposomal irinotecan for total volumes of 2 or 3 mL per CED. During the study, the concentration of gadoteridol was reduced stepwise from 2.0 mM to 0.1 mM with the intention of limiting potential toxicities associated with contrast deposition (**Table 3**).

**Table 3.** CED Treatments and Infusate Volume of Distribution

Subject	CED number	GAD concentration (Mm)	Infusion volume (ml)	Infusion duration (minutes)	Tumor volume (cm^3^)	GAD volume distribution per CED treatment (cm^3^)	Total volume of gad distribution (cm^3^)	Tumor volume covered by gad (cm^3^)	Tumor volume covered by gad (%)
PNOC009-1	1	2.0	2	236	45.1	7.6	7.6	7.5	16.6
PNOC009-2	1	2.0	3	416	23.9	9.8	12.2	10.3	42.9
	2	2.0	3	350		7.2			
PNOC009-3	1	2.0	3	335	26.7	7.9	11.2	10.6	39.7
	2	1.0	2	244		5.8			
PNOC009-4	1	0.5	3	419	21.1	5	16.4	13.5	63.7
	2	0.5	3	359		6.4			
	3	0.5	3	344		6.9			
	4	0.5	3	460		5.5			
	5	0.1	3	377		0.7			
PNOC009-5	1	0.5	3	417	32.4	7.0	11.7	10.1	31.3
	2	0.5	3	387		5.4			
PNOC009-6	1	0.5	3	357	24.4	5.6	5.6	4.4	17.9

Abbreviations: mM = millimolar; mL = milliliter; cm^3^ = cubic centimeter; GAD = Gadoteridol.

**Figure 2.**
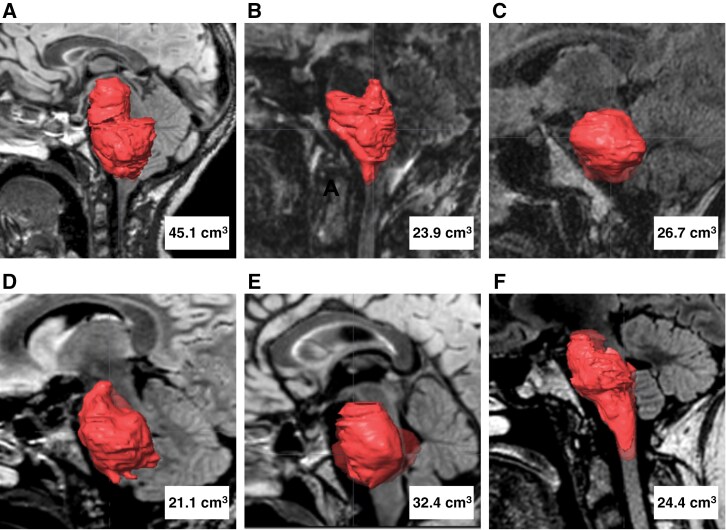
Tumor volumetric study. Tumor volume in cubic centimeters (cm^3^) based on FLAIR imaging prior to treatment for A) PNOC009-1, B) PNOC009-2, C) PNOC009-3, D) PNOC009-4, E) PNOC009-5, F) PNOC009-6.

**Figure 3.**
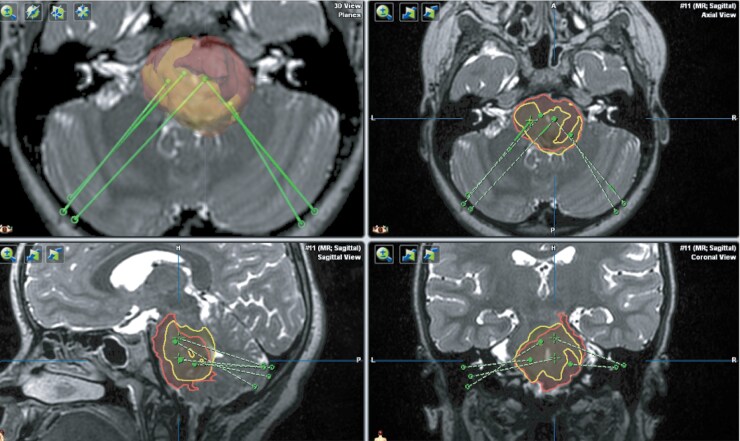
3D T2-weighted MRI overlay of baseline tumor volume and post-CED infusate distribution (PNOC009-4). Tumor volume at baseline (red) and total volume of infusate distribution (yellow) after 5 CED treatments.

The mean tumor volume prior to the first CED treatment based on preoperative MRI FLAIR sequences was 28.9 ± 8.8 cm^3^ as shown in **Figure 2**. Using the T1-weighted images for gadoteridol distribution, the mean tracer volume distribution per CED infusion was 6.2 ± 2.1 cm^3^. The total tumor volume coverage ranged from 16.6% to 63.7% with a mean of 35.4 ± 17.6%. As expected, in patients with multiple CED treatments, a larger volume of tumor coverage was observed. The mean Vd/Vi ratio was 2.6 ± 0.8. Although all catheters did show gadoteridol distribution within the tumor, distribution was limited to local regions while other areas blocked infusate delivery (**Figure 3 and Supplementary****Figure S2**). Leakage out of the tumor, along white matter tracts, was only seen for the supratentorial trajectory. The mean volume of gadoteridol outside of the tumor per patient was 1.4 ± 0.9 cm^3^.

## Discussion

This phase 1 study is the first to investigate the safety, tolerability, and distribution of nal-IRI delivered via CED in patients with DIPG. We show that up to 60 mg of nal-IRI with a maximum total volume of 3 mL per CED was well tolerated. Grade 3 AEs were all neurologic: muscle weakness, dysarthria, and gait disturbance. The patients that experienced dysarthria, and gait disturbance recovered over time. The favorable toxicity profile presented in this study has also been reported by other clinical trials utilizing CED for DIPG patients.^13,14^ In a similar phase 1 study investigating CED of MTX110 (aqueous panobinostat) in patients with DIPG population (PNOC015), we previously reported a very similar toxicity profile.^13^ These AEs were comparable to our current study and included muscle weakness, gait disturbances, and cranial nerve dysfunction. In another phase 1 study, Souweidane et al. investigated CED of the radiolabeled antibody [^124^I]-8H9 and reported no grade 3 or higher treatment-related toxicity.^14^ Given that no DLTs occurred in this study, the MTD was not reached. Such favorable safety profiles are reassuring but do limit the assessment of true MTD. In contrast, most adult early-phase clinical trials investigating CED reach an MTD.^15–22^ One possible distinction is that due to the high-risk anatomical location of the tumor dose escalation in CED for patients with DIPG, dose levels may be overly cautious. Regardless, overall favorable toxicity profiles in several clinical CED trials in DIPG show that this approach is safe and warrants further dose escalation in future studies.

From a design standpoint, PNOC009 implemented an ATD with the goal of treating fewer patients at suboptimal dosing. However, due to grade 2 AEs early on in cycle 1, the design transitioned to a 3 + 3 design, resulting in little benefit of the ATD design. More recent designs such as the Bayesian optimal interval (BOIN), interval 3 + 3, or Bayesian optimal interval phase I/II (BOIN12) for immuno- and targeted therapies might be more suitable and should be implemented in future CED trials.^23,24^

Nal-IRI offers prolonged circulation compared to conventional irinotecan, allowing for sustained release of its active metabolite, SN-38, which mediates its cytotoxic effects^.25,26^ Furthermore, its liposomal formulation, combined with the enhanced permeability and retention effect, facilitates the preferential accumulation of nal-IRI in tumor tissues.^24^ Notably, in this study, no grade 2 or higher AEs were attributed to nal-IRI. As a secondary objective, this study aimed to build on prior work investigating this agent in malignant glial tumors in adults.^27,28^ In PNOC009, OS12 was 67% which is notably higher than previous reports in this patient population.^29^ However, this result should be approached with caution as it may be biased due to limited patient numbers, patient censoring, and resulting lack of power. Additionally, the inclusion of both pediatric and adult DIPG patients may bias the overall outcome findings, given that adult DIPG typically presents a less aggressive clinical course and differences in molecular profile.^3^ Interestingly, one adult patient (PNOC009-2) reported an OS of 91 months at the last study visit. This patient was diagnosed with an IDH-mutant glioblastoma after the study period. Because we did not require histologic confirmation, we cannot comment on the integrated molecular diagnosis of the other patients and the possible associated H3 K27 alterations. Previous reports have shown that ~25% of all diffuse brainstem gliomas are lacking H3 K27M mutations and that imaging characteristics have limited correlation with H3 K27M mutation status.^30–32^ Therefore, to delineate responding patients and comprehend the molecular profile of these patients, future studies should aim to consistently incorporate molecular profiling. Given the concern that biopsies are creating tracks that will limit CED, future CED trials should also consider the assessment of cell-free DNA for diagnosis.

As an exploratory objective, this study modeled the drug distribution via gadoteridol co-infusion. We showed that the mean tumor coverage was 35.4% for all CED treatments combined, and multiple CED treatments led to a larger volume of tumor coverage as expected. This result is lower than the mean tumor coverage of 56.2% seen in our PNOC015 study. The highest total tumor volume coverage combining all treatments was 63.7% for PNOC009-4 who received five CED treatments. This falls within the range of PNOC015 where tumor coverage ranged between 35.6% and 78.5% and is reflective of the lower number of treatment cycles in PNOC009 compared to PNOC015.

Additionally, varying and often low gadoteridol concentrations in this study likely led to the underestimation of the true drug distribution. However, our aim was not to determine the exact extent of distribution but rather to estimate drug distribution and identify potential leakage or pooling. Our findings suggest that certain regions within the tumor may impede drug penetration, indicating that multiple or repeated catheter placements may be necessary to ensure comprehensive coverage. Tissue distribution of the infusate is affected by factors that are not easily measured prior to treatment. In some cases, elevated tumor interstitial pressure can drive the infusate into surrounding areas, including peritumoral tissue. In this study, each infusion followed a newly optimized trajectory, leveraging convective flow to reduce pooling and leakage. This scenario may differ in chronic infusion settings where the catheter remains in place for repeated administrations to the same area.

In this context, it is also essential to acknowledge that in CED procedures utilizing gadolinium-based contrast agents as markers, the contrast agent serves as a surrogate for the therapeutic compound. Emerging imaging techniques, such as chemical exchange saturation transfer (CEST) magnetic resonance imaging could provide more accurate real-time insights into drug localization and tissue uptake.^33^ These advancements could potentially allow for better prediction of therapeutic response and help in adjusting infusion parameters dynamically during treatment. Nonetheless, a previous report showed a volumetric and anatomic overlap between the gadolinium-based contrast agent and simultaneously infused molecule, supporting our notion that gadoteridol serves as a valid proxy for drug distribution in PNOC009.^34^

While this study employed optimized infusion trajectories, additional strategies warrant exploration to further enhance tumor coverage. For instance, alternative infusion regimens, such as chronic CED enabling continuous therapeutic delivery, should be compared with repeated CED protocols to identify the most effective drug administration approach. Furthermore, microenvironment modulation, such as reducing interstitial fluid pressure or enzymatic matrix alteration, can improve convective drug distribution and potentially further enhance the volume of distribution and therapeutic efficacy of CED in brain tumors.^35^

It is important to note that many patients stopped participation early in the study before progression largely due to study burden. Termination of therapy participation was commonly related to the intensity of frequent hospitalizations and stays in the intensive care unit for monitoring, as well as necessary travel to the site of therapy administration. These considerations highlight the significance of assessing the trial burden in advance and incorporating patient input when designing clinical trials. Another limitation of this study is the early termination due to the discontinuation of drug distribution by the providing company. Despite the requirement for additional surgery, which represents a clear disadvantage of repeated CED, the repeated CED treatment employed in this study appears to facilitate conditions that enable convection, improving overall tumor coverage. Additionally, PNOC009 allowed further validation of CED in patients with DIPG, expanding existing knowledge, and showing that repeated CED of nal-IRI has an acceptable safety profile with promising tumor coverage.

In conclusion, CED as a delivery method for nal-IRI is safe and tolerable and holds significant promise for patients with DIPG. This approach should be employed in a larger patient population to confirm an RP2D and therapeutic efficacy.

## Supplementary Material

vdaf093_suppl_Supplementary_Materials

## Data Availability

Data will be made available on reasonable request.
