# Supplementary material for: PNOC009: Convection-enhanced delivery of liposomal irinotecan in patients with newly diagnosed diffuse intrinsic pontine glioma
Source: Neurooncol Adv. 2025 May 13;7(1):vdaf093. doi: 10.1093/noajnl/vdaf093 (PMC12202035; doi:10.1093/noajnl/vdaf093)
Supplement: vdaf093_suppl_Supplementary_Materials [file vdaf093_suppl_supplementary_materials.docx]

**Supplemental Table S1:** inclusion and exclusion criteria

## Inclusion Criteria

Patients must have eligibility evaluations performed within 14 days prior to registration (unless otherwise stated) and must meet all inclusion and none of the exclusion criteria. In addition, the patient must be thoroughly informed about all aspects of the study, including the study visit schedule, required evaluations and all regulatory requirements. The written informed consent must be obtained from the patient or their legal guardian prior to enrollment. The following criteria apply to all patients enrolled onto the study unless otherwise specified. The Inclusion Criteria will be applied **after** patients have completed radiotherapy and within the given time frame per observation as listed below. Enrollment into the trial occurs after completion of radiation therapy and if all eligibility criteria are met.

- Patients with newly diagnosed DIPG by MRI; defined as patients with a pontine location and diffuse involvement of at least 2/3 of the pons are eligible without histologic diagnosis. For lesions with typical imaging features, biopsy is neither encouraged nor required for eligibility. Tumors that are biopsied will be eligible if proven to be supportive of the diagnosis of a DIPG. Consensus of diagnosis by the study team must be met.
- Treatment must begin at a minimum of 4 weeks after, but no later than 14 weeks after, the date of the completion of focal radiotherapy.
- Prior Chemotherapy: Patients should be at least 30 days from last chemotherapy dose prior to start of CED infusion, with exception of antibody half-lives. For antibody therapies, at least 3 half-lives of the antibody after last dose of monoclonal antibody should have passed prior to CED infusion. Patients less than 30 days from last chemotherapy dose should be discussed with the study chair(s).
- Prior Radiation: Patients must have completed prior treatment with standard focal radiotherapy as part of initial treatment for DIPG and had their last dose at least 4 weeks prior to and no later than 14 weeks from the first CED treatment. Patients beyond 14 weeks from radiation therapy but with stable disease should be discussed with the study chair.
- Age ≥ 2 years of age. Patients younger than 3 years of age may be enrolled on study at the discretion of the Study Chair(s) if supporting evidence that brainstem lesion represents a brainstem glioma.
- Karnofsky ≥ 50 for patients > 16 years of age and Lansky ≥ 50 for patients ≤ 16 years of age (see Appendix A). Patients who are unable to walk because of paralysis, but who are up in a wheelchair, will be considered ambulatory for the purpose of assessing the performance score.
- Life expectancy of greater than 12 weeks measured from the date of completion of radiotherapy.
- Corticosteroids: Patients who are receiving dexamethasone must be on a stable or decreasing dose for at least 1 week prior to registration.

Organ Function Requirements

- Adequate Bone Marrow Function Defined as:
- Peripheral absolute neutrophil count (ANC) ≥ 1000/mm^3^ **and**
- Platelet count ≥ 100,000/mm^3^ (transfusion independent, defined as not receiving platelet transfusions for at least 7 days prior to enrollment) **and**
- Adequate Renal Function Defined as:
- Creatinine clearance or radioisotope GFR ≥ 70mL/min/1.73 m^2^ **or**
- A serum creatinine based on age/gender as follows:

| **Age** | **Maximum Serum**  **Creatinine (mg/dL)** | |
| --- | --- | --- |
|  | **Male** | **Female** |
| 3 to < 6 years | 0.8 | 0.8 |
| 6 to < 10 years | 1 | 1 |
| 10 to < 13 years | 1.2 | 1.2 |
| 13 to < 16 years | 1.5 | 1.4 |
| ≥ 16 years | 1.7 | 1.4 |

The threshold creatinine values in this table were derived from the Schwartz formula for estimating GFR utilizing child length and stature data published by the CDC.

- Adequate Liver Function Defined as:
- Bilirubin (sum of conjugated + unconjugated) ≤ 1.5 x upper limit of normal (ULN) for age **and**
- SGPT (ALT) ≤ 110 U/L **and**
- Serum albumin ≥ 2 g/dL.
- Adequate Neurologic Function Defined as:
  - Patients with seizure disorder may be enrolled if on non-enzyme inducing anticonvulsants and well controlled.

- - The effects of irinotecan liposome injection on the developing human fetus are unknown. For this reason women of child-bearing potential and men must agree to use adequate contraception (hormonal or barrier method of birth control; abstinence) prior to study entry, for the duration of study participation and 4 months after completion of irinotecan liposome injection administration. Should a woman become pregnant or suspect she is pregnant while she or her partner is participating in this study, she should inform her treating physician immediately.
  - A legal parent/guardian or patient must be able to understand, and willing to sign, a written informed consent and assent document, as appropriate

**Exclusion Criteria**

- Patients who had clinical and/or radiographic (MRI) progression of tumor following external beam radiation therapy.
- Patients with metastatic disease, including leptomeningeal or subarachnoid disseminated disease.
- Patients with tumor morphology or other imaging findings that predict poor coverage of the majority of the tumor including significant tumor volume outside the pons or presence of large cysts within the tumor that would prevent adequate tumor coverage by CED. Patients with concern for adequate tumor coverage based on tumor morphology should be discussed with the study chairs.
- Patients who are receiving any other tumor-directed therapy.
- Patients with MRI or clinical evidence of uncontrolled tumor mass effect are excluded; the patients should be discusserd with the study chair(s) and study neurosurgeon prior to any planned CED treatment.
- Untreated symptomatic hydrocephalus determined by treating physician.
- Patients should not be on enzyme-inducing anticonvulsants or other drugs that might interact with the cytochrome P450 enzyme system. If previously on an EIAED, patients should be off for at least 10 days prior to CED infusion and discussed with the Study Chair.
- History of allergic reactions attributed to compounds of similar chemical or biologic composition to irinotecan, topotecan, gadolinium, or lipids.
- Uncontrolled intercurrent illness including, but not limited to, ongoing or active infection, symptomatic congestive heart failure, unstable angina pectoris, cardiac arrhythmia, or psychiatric illness/social situations that would limit compliance with study requirements.
- Female patients of childbearing potential must not be pregnant or breast-feeding. Female patients of childbearing potential must have a negative serum or urine pregnancy test within 14 days of registration.
- Patients who are unable to return for follow-up visits or obtain follow-up studies required to assess toxicity to therapy. Telemedicine visits are acceptable.

**Supplemental Table S2**: Summary of All Treatment Related Adverse Events

| **Toxicity** | **Grade 1** | **Grade 2** | **Grade 3** | **Frequency** |
| --- | --- | --- | --- | --- |
| **Pyramidal tract syndrome** | 1 |  |  | 1 |
| **Vomiting** | 6 | 1 |  | 7 |
| **Nausea** | 5 |  |  | 5 |
| **Dysesthesia** | 2 |  |  | 2 |
| **Muscle weakness right-sided** | 1 | 3 | 2 | 6 |
| **Abducens nerve disorder** | 4 | 1 |  | 5 |
| **Dizziness** | 1 | 3 |  | 4 |
| **Hiccups** | 1 |  |  | 1 |
| **Fatigue** | 2 | 3 |  | 5 |
| **Urinary retention** | 1 | 1 |  | 2 |
| **Insomnia** | 2 |  |  | 2 |
| **Constipation** | 6 |  |  | 6 |
| **Lymphocyte count decreased** | 3 | 3 |  | 6 |
| **Creatinine increased** | 1 |  |  | 1 |
| **Hypocalcemia** | 2 |  |  | 2 |
| **Paresthesia** | 3 |  |  | 3 |
| **Oral dysesthesia** | 1 |  |  | 1 |
| **Dysphagia** | 2 | 2 |  | 4 |
| **Ataxia** | 1 | 2 |  | 3 |
| **Hyperglycemia** | 1 |  |  | 1 |
| **Tinnitus** | 3 |  |  | 3 |
| **Gastroesophageal reflux disease** | 1 |  |  | 1 |
| **Muscle weakness left-sided** | 1 |  |  | 1 |
| **Facial muscle weakness** |  | 1 |  | 1 |
| **Facial nerve disorder** | 3 |  |  | 3 |
| **Sinus bradycardia** | 2 |  |  | 2 |
| **Hyponatremia** | 1 |  |  | 1 |
| **Weight loss** | 3 |  |  | 3 |
| **Rash acneiform** | 1 |  |  | 1 |
| **Dry skin** |  | 1 |  | 1 |
| **White blood cell decreased** | 1 |  |  | 1 |
| **Glossopharyngeal nerve disorder** | 1 |  |  | 1 |
| **Hypoglossal nerve disorder** | 2 |  |  | 2 |
| **Headache** | 2 |  |  | 2 |
| **Anorexia** | 1 |  |  | 1 |
| **Conjunctivitis** | 2 | 2 |  | 4 |
| **Accessory nerve disorder** | 2 |  |  | 2 |
| **Nasal congestion** | 1 |  |  | 1 |
| **Hypokalemia** | 2 |  |  | 2 |
| **Fever** | 1 |  |  | 1 |
| **Fall** | 2 |  |  | 2 |
| **Dyspnea** | 1 |  |  | 1 |
| **Gait disturbance** |  | 4 | 1 | 5 |
| **Dysarthria** | 2 | 3 | 1 | 6 |
| **Scalp pain** |  | 1 |  | 1 |
| **Eye disorders - Other** |  | 1 |  | 1 |

**Figure S1**: Dose Escalation Scheme


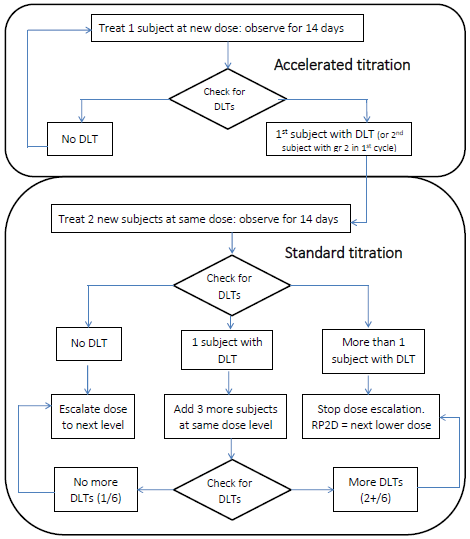


Abbreviation: DLT – Dose-limiting toxicity

**Figure S2:** Visualization of Virtual Trajectory and Tumor Overlay on Axial 3D T1-Weighted MR Images Pre- and Post-CED Infusion for **A)** PNOC009-01 first infusion **B)** PNOC009-02 first infusion **C)** PNOC009-02 second infusion **D)** PNOC009-03 first infusion **E)** PNOC009-03 second infusion **F)** PNOC009-04 first infusion **G)** PNOC009-04 second infusion **H)** PNOC009-04 third infusion **I)** PNOC009-04 fourth infusion **J)** PNOC009-04 fifth infusion **K)** PNOC009-05 first infusion **L)** PNOC009-05 second infusion **M)** PNOC009-06 first infusion


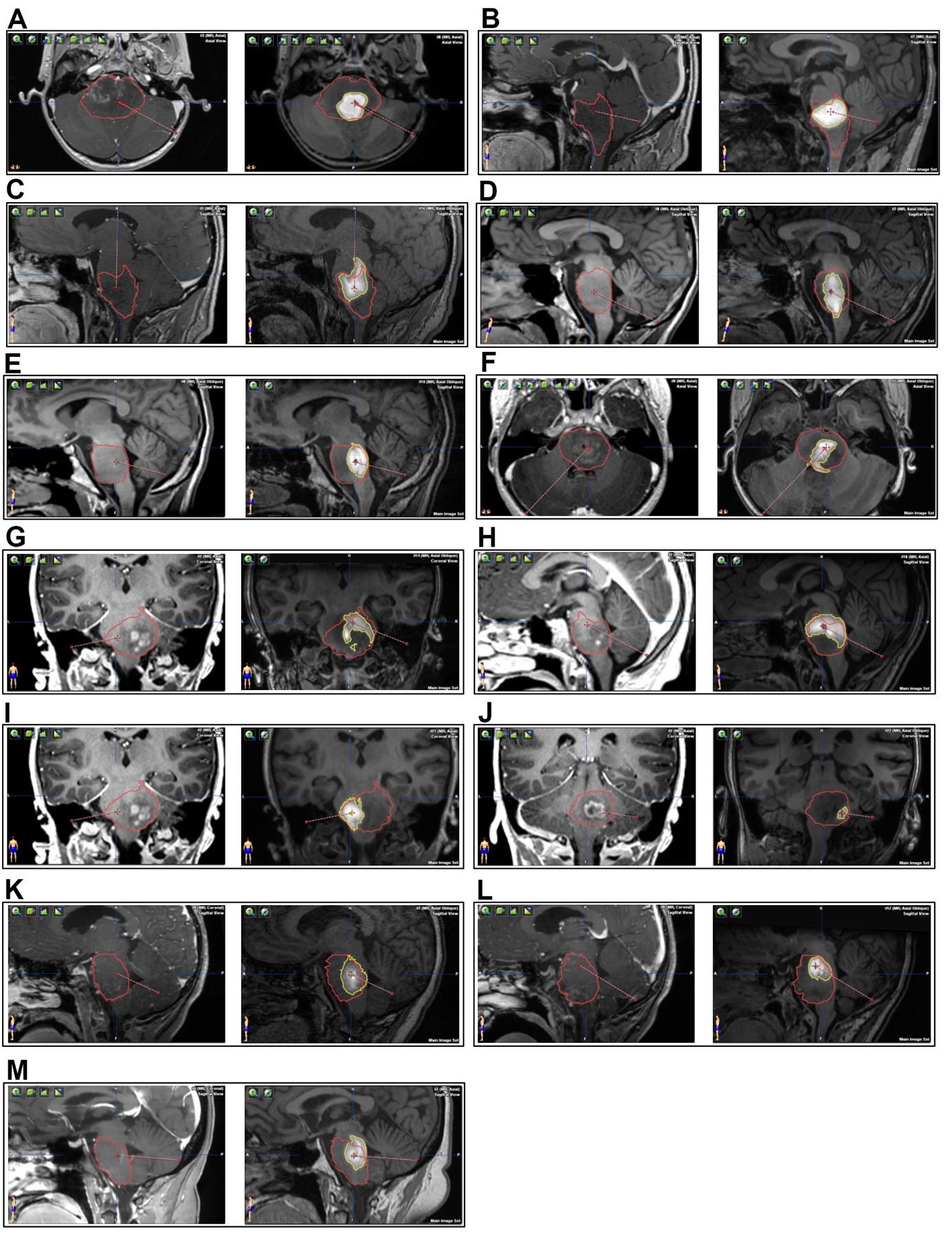


Red dashed lines = virtual trajectory); red outline = tumor; yellow outline = distribution after nal-IRI/gadoteridol infusion.
